# Amplification and overexpression of the EGF receptor and c-erbB-2 proto-oncogenes in human stomach cancer.

**DOI:** 10.1038/bjc.1991.243

**Published:** 1991-07

**Authors:** N. R. Lemoine, S. Jain, F. Silvestre, C. Lopes, C. M. Hughes, E. McLelland, W. J. Gullick, M. I. Filipe

**Affiliations:** ICRF Molecular Oncology Group, Hammersmith Hospital, London, UK.

## Abstract

**Images:**


*Br. J. Cancer* (1991), **64**, 79–83 © Macmillan Press Ltd., 1991

### Amplification and overexpression of the EGF receptor and c-*erb*B-2 proto-oncogenes in human stomach cancer

N.R. Lemoine[1], S. Jain[2], F. Silvestre[3], C. Lopes[4], C.M. Hughes[1], E. McLelland[1], W.J. Gullick[1] & M.Isabel Filipe[2]

[1]*ICRF Molecular Oncology Group, MRC Cyclotron Building, Hammersmith Hospital, Du Cane Road, London W12 0HS;* [2]*Department of Histopathology, UMDS Guy's Hospital, London Bridge, London SE1 9RT, UK;* [3]*Department of Histopathology, Hospital Sto Antonio, Porto, Portugal; and* [4]*Department of Histopathology, Institute Portugues de Oncologia, Porto, Portugal.*

Classification of stomach cancer by conventional pathological criteria has not helped in the assessment of prognosis of this tumour (Morson *et al*., 1990) and the availability of new markers that could be applied to routinely-processed pathologic material would be of great value. Recent studies have demonstrated that assessment of gene copy number and expression levels of the receptor tyrosine kinase oncogenes encoding the EGF receptor (also known as c-*erb*B) and c-*erb*B-2 (also known as *HER*2 or *neu*) can offer useful information for the prediction of prognosis in several common human cancers, such as breast cancer and ovarian cancer. In stomach cancer, amplification or overexpression of c-*erb*B-2 has been reported in 19–40% of cases (Tal *et al*., 1988; Yokota *et al*., 1988; Park *et al*., 1989; Oda *et al*., 1990; Gutman *et al*., 1989; Falck & Gullick, 1989; Houldsworth *et al*., 1990) but only one study has so far examined the concordance between level of expression of this oncogene determined by immunohistochemistry in routinely-processed pathology specimens and gene copy number determined by Southern blotting (Houldsworth *et al*., 1990). Until recently, antibodies which reliably detect EGF receptor expression in fixed and paraffin-embedded pathological material have not been available, limiting the study of receptor protein expression. Overexpression of EGF receptors has been detected in frozen samples of gastric adenocarcinoma at a frequency of up to 35% in advanced disease (Yasui *et al*., 1988*a*; *b*). However, amplification of the EGF receptor gene appears to be uncommon in stomach cancer (Yoshida *et al*., 1989). We set out to determine the frequency of EGF receptor and c-*erb*B-2 gene amplification and rearrangement by Southern blotting, and to survey the expression of each of these proto-oncogenes by immunohistochemistry in a parallel series of frozen and routinely-fixed paraffin-embedded gastric cancer specimens.

Gastrectomy specimens were examined by a pathologist immediately after resection and samples of cancer (confirmed on frozen section) were excised, snap-frozen in liquid nitrogen and stored below −70°C until required. The remainder of each specimen was then fixed in formalin or Bouin's fixative and processed for embedding in paraffin blocks. Peripheral blood samples were taken from each patient and the white cell fraction kept frozen below −70°C until required.

High molecular weight DNA was extracted from tumour samples and, in 30 out of the total of 40 cases, a paired sample of peripheral blood leucocytes using a *Model 340A Nucleic Acid Extractor* from Applied Biosystems, EcoRI-digested DNA (10 µg) was electrophoresed in 0.8% agarose gels and transferred to Hybond-N filters (Amersham). Hybridisation and washing were carried as recommended by the manufacturer. Hybridisation probes used to detect proto-oncogene sequences were, for detection of the EGF receptor gene, a 1.8 kb EcoRI purified fragment of p64.1 (Ullrich *et al*., 1984) and for detection of c-*erb*B-2, a 3.25 kb *Hind*III/*Kpn*I fragment of pSV$_2$*erb*B-2 which contains the complete cDNA of c-*erb*B-2 (Yamamoto *et al*., 1986). The blots were stripped and then rehydridised with a 1.8 kb *Pst*I fragment of beta-actin (Cleveland *et al*., 1980) to control for loading differences between samples.

Signal intensity on autoradiographs was quantitated using an *Ultroscan XL* laser densitometer (LKB).

Polymerase chain reaction (PCR) amplification and oligonucleotide probing for transmembrane mutations of the c-*erb*B-2 proto-oncogene was carried out using oligonucleotides and protocols as previously described (Lemoine *et al*., 1990*a*).

To detect c-*erb*B-2 immunoreactivity in frozen and paraffin sections an affinity-purified preparation of rabbit antibody 21N (Gullick *et al*., 1987) was used at a concentration of 2.6 $\mu g\,ml^{-1}$. This reagent has been well characterised and widely used for immunohistochemical detection of c-*erb*B-2 in tissue sections (Gusterson *et al*., 1988). To detect EGF receptor immunoreactivity in frozen and paraffin sections an affinity-purified preparation of the rabbit antibody 12E (raised against a synthetic peptide representing residues 1059 to 1072 in the cytoplasmic domain of EGF receptor, Gullick *et al*., 1985) was used at a concentration of 4 $\mu g\,ml^{-1}$, as well as an affinity-purified preparation of the rabbit antibody 14E (raised against a synthetic peptide of residues 1164 to 1176 of EGF receptor, Gullick *et al*., 1985) at 4 $\mu g\,ml^{-1}$. The monoclonal antibody EGFR1 (raised against A431 cells and recognises the native folded external domain of EGF receptor, Waterfield *et al*., 1982; has been extensively characterised for use in immunohistochemistry, Gullick *et al*., 1986) was used at a concentration of 10 $\mu g\,ml^{-1}$ to detect EGF receptor immunoreactivity in frozen sections only.

Paraffin sections were rehydrated by passage through xylene and graded alcohols to phosphate-buffered saline. Endogeneous peroxidase activity was quenched by incubation in 0.3% hydrogen peroxide for 30 min and then rinsing in distilled water. For frozen sections endogenous peroxidase activity was quenched by incubation in 0.1% phenylhydrazine hydrochloride for 5 min and then rinsing in distilled water. The immunohistochemical technique involved the sequential application of the following reagents: the primary antibody (21N, 12E, 14E or EGFR1) at the concentration stated above for 1 h at room temperature, a secondary biotinylated anti-rabbit (for 21N, 12E and 14E) or anti-mouse (for EGFR1) antibody (DAKOPATTS) diluted 1:500 for 30 min, and ABComplex/HRP (DAKOPATTS) for 30 min. Each incubation was followed by rinsing the tissue sections in phosphate-buffered saline three times (5 min each). The sites of immunoprecipitate were identified by light microscopy following treatment with a chromogen, 3,3′-diaminobenzidine. No special treatments of the section were required for use of these antibodies. A standard avidin-biotin-peroxidase complex method was used as previously described (Falck & Gullick, 1989). Specificity of the antibodies was tested by loss

Correspondence: N. Lemoine, ICRF Molecular Oncology Group, MRC Cyclotron Building, Hammersmith Hospital, Du Cane Road, London W12 0HS. UK.
Received 8 January 1991; and in revised form 28 February 1991.

of staining after pre-absorption of antibody with the immunising peptide, and retention of staining after pre-absorption with other non-specific peptides. Overexpression of the growth factor receptor (EGF receptor or c-*erb*B-2) is defined as staining of tumour cell membranes, often accompanied by cytoplasmic staining. As detailed below, such immunoreactivity was not observed on cell membranes in normal stomach mucosa.

The results of tumour analysis are presented in Tables I and II. In the normal stomach very faint cytoplasmic immunoreactivity of EGF receptor was observed only in the parietal cells of the gastric glands when using the 12E antibody. With all of the anti-EGF receptor antibodies there was strong staining of the brush border of surface enterocytes of the normal small intestine and in intestinal metaplasia of the stomach (Figure 2b). Immunoreactivity for c-*erb*B-2 was not detected in non-neoplastic stomach with the antibody 21N at the concentration used.

In the tumour series, overexpression of the c-*erb*B-2 growth factor receptor proto-oncogene (recognised as staining of tumour cell membranes) was detected in 26% of all the cases examined in paraffin section, more frequently in the intestinal type (53%) than in diffuse type (8%). There was no apparent association with tumour stage or lymph node involvement, nor with tumour site or growth pattern (expanding/infiltrative). Southern blot analysis revealed four cases of c-*erb*B-2 gene amplification (Figure 1a) which represents a frequency of 13% in the 30 cases examined. Three of these cases were classified as intestinal type tumours and one as diffuse type by the Lauren classification. Strong membrane immunoreactivity with the 21N antiserum was demonstrable in both frozen sections and paraffin sections, usually with more than 80% of the tumour cells positive in these gene-amplified cases (Table II). In four other cases, the c-*erb*B-2 gene was present in apparently single copy on Southern blot analysis and there was moderate to strong membrane immunoreactivity just in some areas of the tumour sections. Two cases (omitted from Table II for clarity) were not examined by Southern blot, but had focal 21N immunoreactivity (less than 5% of tumour cells positive). The failure to identify immunoreactivity in frozen sections of some tumours in which there was definite positivity in paraffin sections could be due to the patchy nature of overexpression of c-*erb*B-2 in cases without gene amplification. The sensitivity of immunohistochemical detection of c-*erb*B-2 overexpression appears to be at least as good in paraffin sections as in frozen sections in this series. However, other authors have shown that, at least in breast cancer, low levels of c-*erb*B-2 immunoreactivity detectable in frozen material may not survive exposure to fixation and processing for paraffin embedding (Slamon *et al.*, 1989).

Oligonucleotide probing of PCR-amplified genomic DNA did not detect any evidence of potentially activating mutations in the transmembrane region of the c-*erb*B-2 oncogenes in 31 cases examined. This is consistent with the absence of this potential mechanism of activation in other human cancers we have examined including 100 breast cancers, 60 thyroid tumours, 23 pancreatic cancers and 32 brain tumours (Lemoine *et al.*, 1990*a*, *b*; Hall *et al.*, 1990; Tuzi *et al.*, 1991).

Overexpression of EGF receptor (recognised as staining of tumour cell membranes often accompanied by cytoplasmic staining) was detected in 18% of gastric cancers examined by immunohistochemistry on paraffin sections and, as for c-*erb*B-2, was more frequent in intestinal type tumours (27%) than in diffuse type tumours (12%). There was no apparent association with tumour stage or lymph node involvement, nor with tumour site or growth pattern (expanding/infiltrative). Southern blot analysis revealed two cases (cases 4 and 5 illustrated in Figure 1b) of EGF receptor gene amplification out of 30 cases analysed, but only in one of these was overexpression demonstrable as uniform (>80% of the tumour positive) membrane immunoreactivity with the 12E and 14E antibodies in frozen and paraffin sections (Figure 2a), and the EGFR1 antibody in frozen sections. The explanation for our failure to demonstrate overexpression in the other case is presently unknown. There were seven cases of EGF receptor overexpression in the absence of gene amplification, but in these cases the immunoreactivity was not uniform and some areas of the tumours were completely negative. The immunoreactivity for EGF receptor in these cases was demonstrable with all of the antibodies used in frozen section, but while the EGFR1 and 12E antibodies gave comparable results in the detection of membrane expression, the 14E antibody gave consistently weaker staining and often showed cytoplasmic rather than membrane localisation in the tumour cells. The sensitivity of immunohistochemical detection of EGF receptor overexpression using the 12E antiserum was higher in paraffin sections than in frozen sections of this series of stomach cancers. This is at least partly accounted for by the greater ease of interpretation of EGF receptor immunoreactivity, often accentuated at the

**Table II** Extent of immunoreactivity in cases of EGFR or c-*erb*B-2 overexpression

| *Immunoreactivity*[a] *in paraffin section* | *EGF receptor* *Gene amplification (n = 2)* | *Single copy (n = 7)* | *c-erbB-2* *Gene amplification (n = 4)* | *Single copy (n = 4)* |
|---|---|---|---|---|
| Uniform (>80% tumour) | 1 | | 3 | |
| Patchy (20–80% tumour) | | 6 | 1 | 1 |
| Patchy (5–20% tumour) | | | | 2 |
| Focal (<5% tumour) | | 1 | | 1 |
| Negative | 1 | | | |

[a]Immunoreactivity here refers to definite staining of the tumour cell membranes, which was usually accompanied by some cytoplasmic staining. Cases in which there was only cytoplasmic staining are not included here.

**Table I** Frequency of abnormalities[a] of EGF receptor and c-*erb*B-2 in human gastric cancers

| | *Lauren classification* *Intestinal* | *Diffuse* | *WHO classification* *Well/moderately differentiated tubular/papillary* | *Poorly differentiated tubular/papillary* | *Signet ring/ mucoid* | *Undifferentiated* |
|---|---|---|---|---|---|---|
| EGFR | | | | | | |
| Frozen sections | 3/16 (19%) | 2/24 (8%) | 3/16 (19%) | 2/19 (11%) | 0/4 | 0/1 |
| Paraffin sections | 4/15 (27%) | 3/24 (12%) | 4/16 (25%) | 3/19 (16%) | 0/4 | 0/1 |
| Gene amplification | 1 case, >20 fold | 1 case, >20 fold | 1 case, >20 fold | 1 case, >20 fold | | |
| c-*erb*B-2 | | | | | | |
| Frozen sections | 6/16 (38%) | 1/24 (4%) | 4/16 (25%) | 1/19 (5%) | 2/4 | 0/1 |
| Paraffin sections | 8/15 (53%) | 2/24 (8%) | 5/16 (31%) | 3/19 (16%) | 2/4 | 0/1 |
| Gene amplification | 1 case, 5–10 fold<br>1 case, 15–20 fold<br>1 case, >20 fold | 1 case, >20 fold | 1 case, 5–10 fold<br>1 case, 15–20 fold | 1 case, >20 fold | 1 case, >20 fold | |

[a]Abnormalities of gene expression are defined as definite membrane staining on immunohistochemistry.

**Figure 1** **a**, Southern blot analysis of c-*erb*B-2 and beta actin genes in gastric adenocarcinomas. Ten micrograms of EcoRI-digested DNA was electrophoresed in an 0.8% agarose gel and transferred to a Hybond-N filter and hybridised with a specific probe, washed under stringent conditions and autoradiographed against Fuji RX film with intensifying screens at −70°C for 3 days. The upper panel shows the filter hybridised with the c-*erb*B-2 probe, and the lower panel shows the same filter after stripping and rehybrisation with a beta actin probe. Numerals over pairs of lanes indicate case numbers (so cases 3 and 4 are illustrated in **a** and **b**), T = tumour DNA, N = normal DNA from peripheral blood. The c-*erb*B-2 proto-oncogene is amplified 15- to 20-fold in case 1, and 5- to 10-fold in case 2. **b**, Southern blot analysis of EGF receptor and beta actin genes in gastric adenocarcinomas. The same filter as in **a** is shown, now hybridised with the EGF receptor probe (upper panel) or beta actin probe (lower panel) using the same conditions described in **a**. The EGF receptor gene is amplified more than 20-fold in both cases 4 and 5.

**Figure 2** **a**, Strong immunoreactivity for EGF receptor recognised by 12E antiserum on the brush border on the luminal surface of enterocytes in intestinal metaplasia of the stomach (right side of figure). The normal columnar epithelial cells of the gastric pits (left side of figure) shows no immunoreactivity. (Formalin-fixed, paraffin section stained by avidin-biotin-peroxidase complex method, haematoxylin counterstain, × 125). **b**, Overexpression of the EGF receptor in a well-differentiated gastric adenocarcinoma of intestinal type. There is a very strong membrane immunoreactivity in all the tumour cells (avidin-biotin-peroxidase complex method, haematoxylin counterstain, × 125).

luminal surface of cells forming tubules and papillary structures, in the paraffin section. The 14E antibody gave positive staining in the same cases as the 12E antibody, but it was weaker and, as noted in frozen sections, it was often cytoplasmic rather than membrane staining. Thus the 12E antibody is likely to be the most useful reagent for the detection of EGF receptor expression in paraffin-embedded material, but application of the 14E antibody (which recognises a different epitope) in parallel can help to confirm the specificity of immunoreactivity.

Only in two cases was there simultaneous overexpression of both proto-oncogenes (we would expect 1.8 cases to show concurrent overexpression of EGF receptor and c-*erb*B-2 by chance on the basis of frequency of each event alone in this small series). One case was a poorly differentiated diffuse type cancer with lymph node metastases which had amplification of the EGF receptor gene and normal copy number of the c-*erb*B-2 gene. The other case was a well differentiated intestinal type cancer with lymph node metastases which had normal copy number of both genes.

Our results are consistent with the findings of other published studies on small series of stomach cancers. One group has reported two studies (Yasui *et al.*, 1988*a*, *b*) in which they found a low prevalence of overexpression of EGF receptor in early disease (4%) but a much higher occurrence in advanced disease (35%). Another study reported a high prevalence of EGF receptor mRNA expression in malignant *vs*

adjacent non-malignant stomach (Bennett *et al.*, 1989). EGF receptor gene amplification appears to be rare in gastric adenocarcinomas, one group reporting only 3% of cases containing additional gene copies (Yoshida *et al.*, 1989; Oda *et al.*, 1990). c-*erb*B-2 is overexpressed in 20% of advanced stomach adenocarcinomas but not in early disease (Yokota *et al.*, 1986; Fukushige *et al.*, 1986; Yokota *et al.*, 1988; Tal; *et al.*, 1988; Park *et al.*, 1989; Falck & Gullick, 1989). It has been reported as more prevalent in tubular adenocarcinomas than in diffuse cancers. Overexpression is generally a consequence of gene amplification but some overexpressing cases may possess a normal gene copy number, and it is intriguing that one group has recently detected overexpression of a protein that binds to the TATA box of the c-*erb*B-2 promoter in such a case (Kameda *et al.*, 1990). Unlike breast cancer, where most tumour cells in a positive tumour uniformly overexpress the protein, in stomach cancer the expression can be patchy or very focal (Falck & Gullick, 1989). This suggests that overexpression may occur later in the progression of stomach cancer than in breast cancer. Gene rearrangement has also been reported at low frequency in gastric cancer (Park *et al.*, 1989). One study of 260 stomach cancers reported that c-*erb*B-2-positive cases behaved more aggressively and had worse prognosis than negative cases (Yonemura *et al.*, 1991).

There is evidence that assessment of EGF receptor and c-*erb*B-2 abnormalities can be useful indicators of prognosis in some other human cancers. For instance, poor clinical outcome has been correlated with amplification and overexpression of the c-*erb*B-2/HER-2/*neu* oncogene in breast cancer (Gullick *et al.*, 1991) and ovarian cancer (Slamon *et al.*, 1989), and of the c-*erb*B/EGF receptor oncogene in breast cancer (Sainsbury *et al.*, 1987). In breast cancer, cases in which there is overexpression of both of these oncogenes have a worse prognosis than those in which only c-*erb*B-2 or the EGF receptor alone is overexpressed (Harris *et al.*, 1989).

We conclude that abnormal expression of the EGF receptor and c-*erb*B-2 proto-oncogene occurs at a significant frequency in gastric cancer, in the presence or apparent absence of gene amplification. We report the application of anti-peptide antibodies which reliably detect such overexpression in routinely-fixed and paraffin-embedded pathologic material and which would be suitable for use in the retrospective analysis of archival material. We suggest that this would be a particularly worthwhile exercise for gastric cancer because conventional typing by the Lauren, WHO and other classifications has been largely unhelpful for prediction of prognosis (Morson *et al.*, 1990). An analysis of the expression of these proto-oncogenes can now be carried out in a larger retrospective series of gastric cancers for which survival data is available.

The authors gratefully acknowledge the generous support of the Imperial Cancer Research Fund.

### References

BENNETT, C., PATERSON, I.M., CORBISHLEY, C.M. & LUQMANI, Y.A. (1989). Expression of growth factor and epidermal growth factor receptor encoded transcripts in human gastric tissues. *Cancer Res.*, **49**, 2104.

CLEVELAND, D.W., LOPATA, M.A., MACDONALD, R.J., COWAN, N.J., RUTTER, W.J. & KIRSCHNER, M.W. (1980). Number and evolutionary conservation of alpha- and beta-tubulin and cytoplasmic beta- and gamma-actin genes using specific cloned cDNA probes. *Cell*, **20**, 95.

FALCK, V.G. & GULLICK, W.J. (1989). c-*erb*B-2 oncogene product staining in gastric adenocarcinoma. An immunohistochemical study. *J. Pathol.*, **159**, 107.

FUKUSHIGE, S.-I., MATSUBARA, K.-I., YOSHIDA, M. & 5 others (1986). Localisation of a novel v-*erb*B-related gene, c-*erb*B-2, on human chromosome 17 and its amplification in a gastric cancer cell line. *Mol. Cell. Biol.*, **6**, 955.

GULLICK, W.J., DOWNWARD, J. & WATERFIELD, M.D. (1985). Antibodies to the autophosphorylation sites of the epidermal growth factor receptor protein-tyrosine kinase as probes of structure and function. *EMBO J*, **4**, 2869.

GULLICK, W.J., MARSDEN, J.J., WHITTLE, N., WARD, B., BOBROW, L. & WATERFIELD, M.D. (1986). Expression of epidermal growth factor receptors on human cervical, ovarian, and vulval carcinomas. *Cancer Res.*, **46**, 285.

GULLICK, W.J., BERGER, M.S., BENNETT, P.L., ROTHBARD, J.B. & WATERFIELD, M.D. (1987). Expression of the c-*erb*B-2 protein in normal and transformed cells. *Int. J. Cancer*, **40**, 246.

GULLICK, W.J., LOVE, S.B., WRIGHT, C. & 4 others (1991). c-*erb*B-2 protein overexpression in breast cancer is a risk factor in patients with involved and uninvolved lymph nodes. *Br. J. Cancer*, **63**, 434.

GUSTERSON, B.A., GULLICK, W.J., VENTER, D.J. & 5 others (1988). Immunohistochemical localization of c-*erb*B-2 in human breast carcinomas. *Mol. Cell. Probes*, **2**, 383.

GUTMAN, M., RAVIA, Y., ASSAF, D., YAMAMOTO, T., ROZIN, R. & SHILOH, Y. (1989). Amplification of c-*myc* and c-*erb*B-2 proto-oncogenes in human solid tumors: frequency and clinical significance. *Int. J. Cancer*, **44**, 802.

HALL, P.A., HUGHES, C.M., STADDON, S.L., RICHMAN, P.A., GULLICK, W.J. & LEMOINE, N.R. (1990). The c-*erb*B-2 proto-oncogene in human pancreatic cancer. *J. Pathol.*, **161**, 195.

HARRIS, A.L., NICHOLSON, S., SAINSBURY, J.R.C., FARNDON, J. & WRIGHT, C. (1989). Epidermal growth factor receptors in breast cancer: association with early relapse and death, poor response to hormones and interactions with *neu*. *J. Steroid Biochem.*, **34**, 123.

HOULDSWORTH, J., CORDON-CARDO, C., LADANYI, M., KELSEM, D.P. & CHAGANTI, R.S.K. (1990). Gene amplification in gastric and esophageal adenocarcinomas. *Cancer Res.*, **50**, 6417.

KAMEDA, T., YASUI, W., YOSHIDA, K. & 5 others (1990). Expression of *erb*B2 in human gastric carcinomas: relationship between p185$^{erbB2}$ expression and gene amplification. *Cancer Res.*, **50**, 8002.

LEMOINE, N.R., STADDON, S.L., DICKSON, C., BARNES, D.M. & GULLICK, W.J. (1990*a*). Absence of activating transmembrane mutations in the c-*erb*B-2 proto-oncogene in human breast cancer. *Oncogene*, **5**, 237.

LEMOINE, N.R., WYLLIE, F.S., LILLEHAUG, J.R. & 8 others (1990*b*). Absence of abnormalities of the c-*erb*B and c-*erb*B-2 oncogenes in human thyroid neoplasia. *Eur. J. Cancer*, **26**, 777.

MORSON, B.C., DAWSON, I.M.P., DAY, D.W., JASS, J.R., PRICE, A.B. & WILLIAMS, G.T. (1990). *Morson & Dawson's Gastrointestinal Pathology*, 3rd Ed. Blackwell, Oxford.

ODA, N., TSUJINO, T., TSUDA, T. & 4 others (1990). DNA ploidy pattern and amplification of ERBB and ERB2 genes in human gastric carcinomas. *Virchows. Archiv. B Cell Pathol.*, **58**, 273.

PARK, J.-B., RHIM, J.S., PARK, S.-C., KIMM, S.-W. & KRAUS, M.H. (1989). Amplification, overexpression, and rearrangement of the *erb*B-2 proto-oncogene in primary human stomach carcinomas. *Cancer Res.*, **49**, 6605.

SAINSBURY, J.R.C., FARNDON, J.R., NEEDHAM, G.K., MALCOLM, A.J. & HARRIS, A.L. (1987). Epidermal growth factor receptor status as predictor of early recurrence of and death from breast cancer. *Lancet*, **i**, 1398.

SLAMON, D.J., GODOLPHIN, W., JONES, L.A. & 8 others (1989). Studies of the HER-2/*neu* proto-oncogene in human breast and ovarian cancer. *Science*, **244**, 707.

TAL, M., WETZLER, M., JOSEFBERG, Z. & 7 others (1988). Sporadic amplification of the *HER2/neu* proto-oncogene in adenocarcinomas of various tissues. *Cancer Res.*, **48**, 1517.

TUZI, N., VENTER, D., KUMAR, S., STADDON, S.L., LEMOINE, N.R. & GULLICK, W.J. (1991). Expression of growth factor receptors in human brain tumours. *Br. J. Cancer*, **63**, 227.

ULLRICH, A., COUSSENS, L., HAYFLICK, J.S. & 12 others (1984). Human epidermal growth factor receptor cDNA sequence and aberrant expression of the amplified gene in A431 epidermoid carcinoma cells. *Nature*, **309**, 418.

WATERFIELD, M.D., MAYES, E.L.V., STROOBANT, P. & 5 others (1982). A monoclonal antibody to the human epidermal growth factor receptor. *J. Cell Physiol.*, **20**, 149.

YAMAMOTO, T., IKAWA, S., AKIYAMA, T. & 5 others (1986). Similarity of protein encoded by the human c-*erb*B-2 gene to epidermal growth factor receptor. *Nature*, **319**, 230.

YASUI, W., HATA, J., YOKOZAKI, H. & 4 others (1988*a*). Interaction between epidermal growth factor and its receptor in progression of human gastric carcinoma. *Int. J. Cancer*, **41**, 211.

YASUI, W., SUMIYOSHI, H., HATA, J. & 4 others (1988*b*). Expression of epidermal growth factor receptor in human gastric and colonic carcinomas. *Cancer Res.*, **48**, 137.

YOKOTA, J., YAMAMOTO, T., TOYOSHIMA, K. & 4 others (1986). Amplification of c-*erb*B-2 oncogene in human adenocarcinomas *in vivo*. *Lancet*, **i**, 765.

YOKOTA, J., YAMAMOTO, T., MIYAJIMA, N. & 6 others (1988). Genetic alterations of the c-*erb*B-2 oncogene occur frequently in tubular adenocarcinoma of the stomach and are often accompanied by amplification of the v-*erb*A homologue. *Oncogene*, **2**, 283.

YONEMURA, Y., NINOMIYA, I., YAMAGUCHI, A. & 7 others (1991). Evaluation of immunoreactivity for c-*erb*B-2 protein as a marker of poor short-term prognosis in gastric cancer. *Cancer Res.*, **51**, 1034.

YOSHIDA, K., TSUDA, T., MATSUMURA, T. & 4 others (1989). Amplification of epidermal growth factor receptor (EGFR) gene and oncogenes in human gastric carcinomas. *Virchows. Archiv. B Cell Pathol.*, **57**, 285.


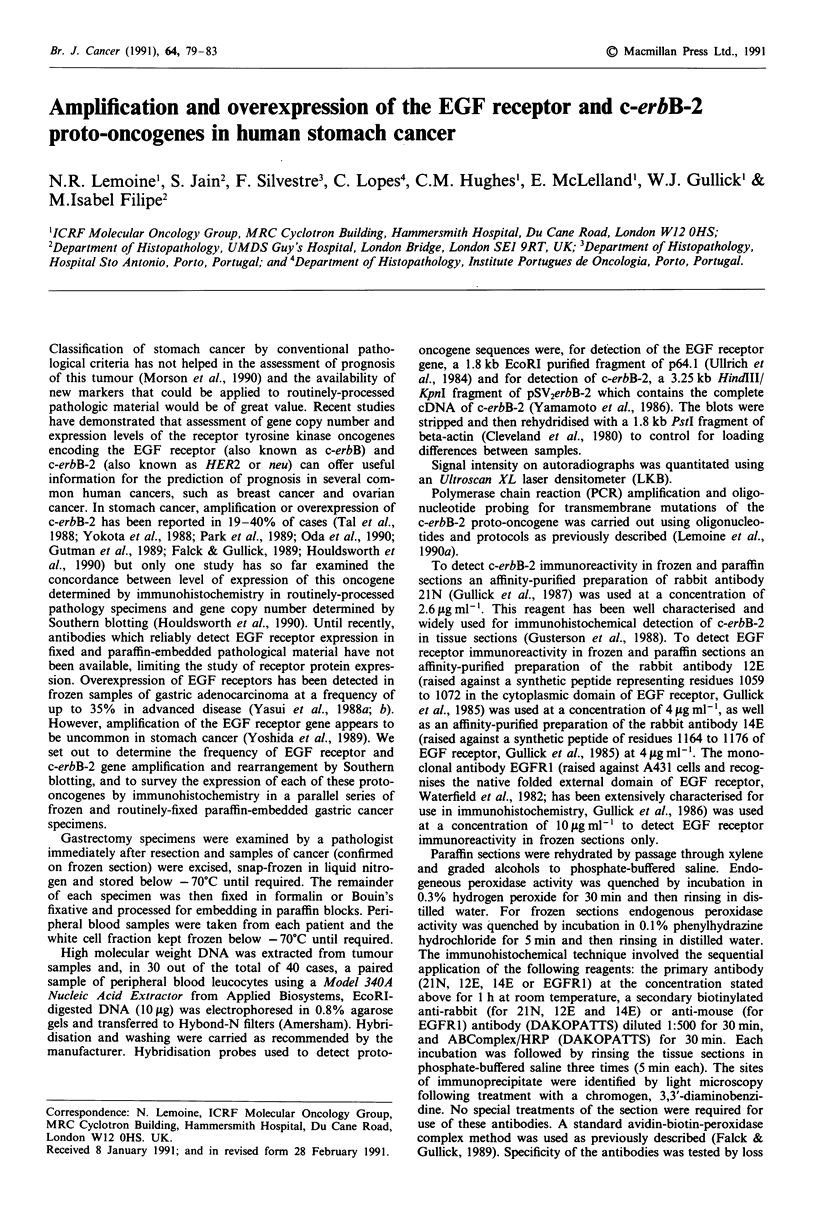

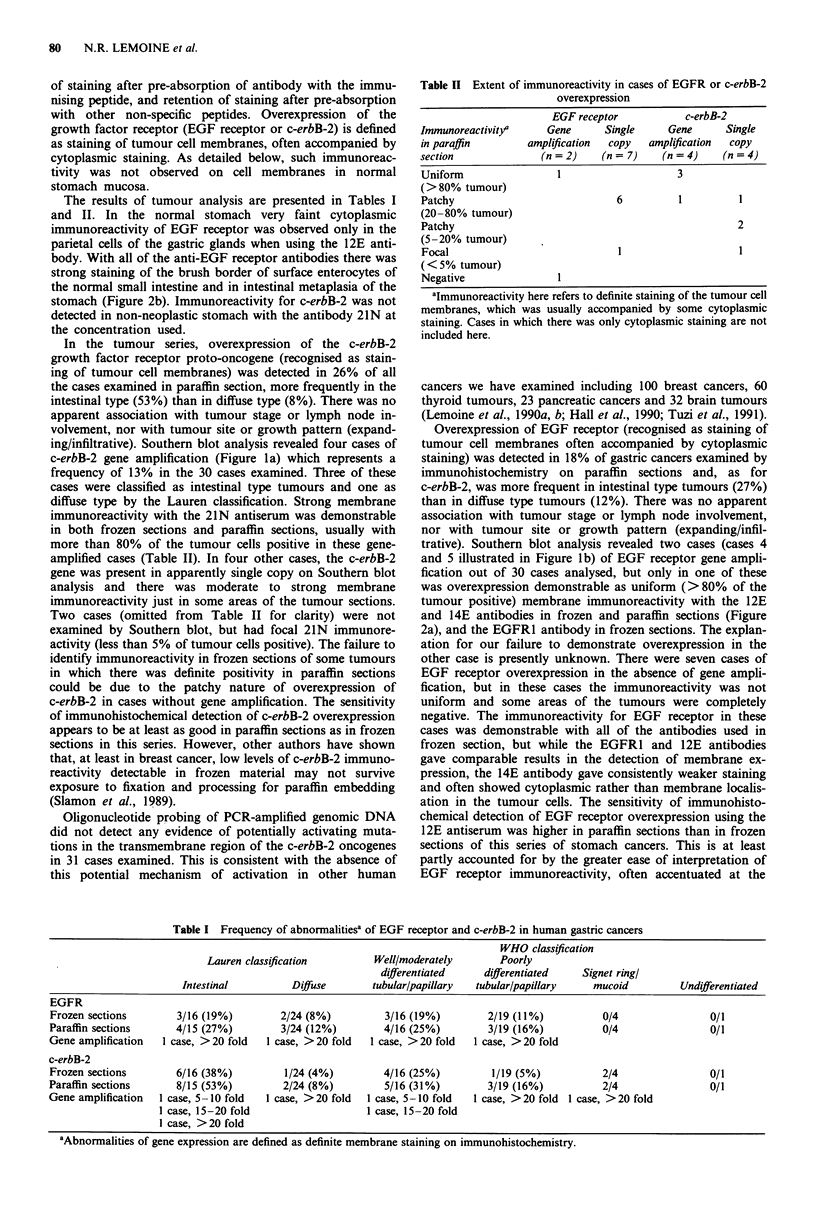

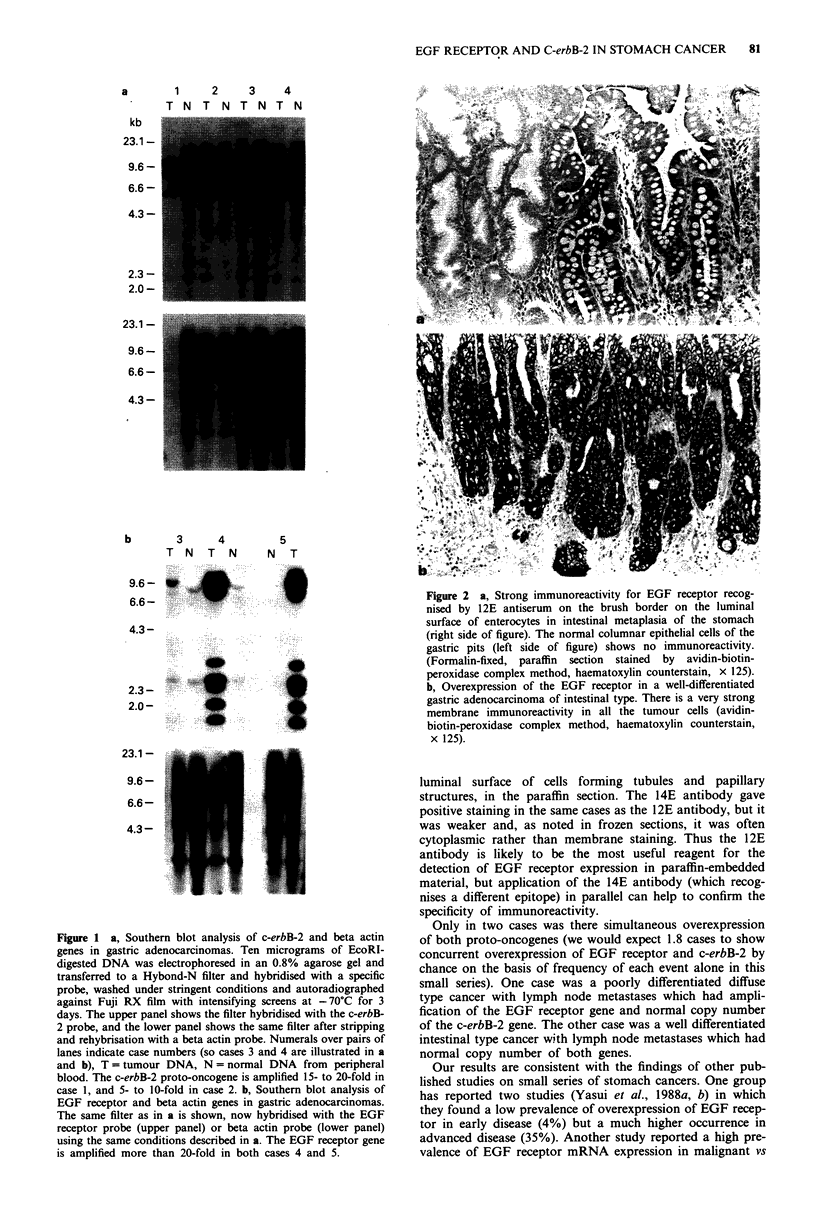

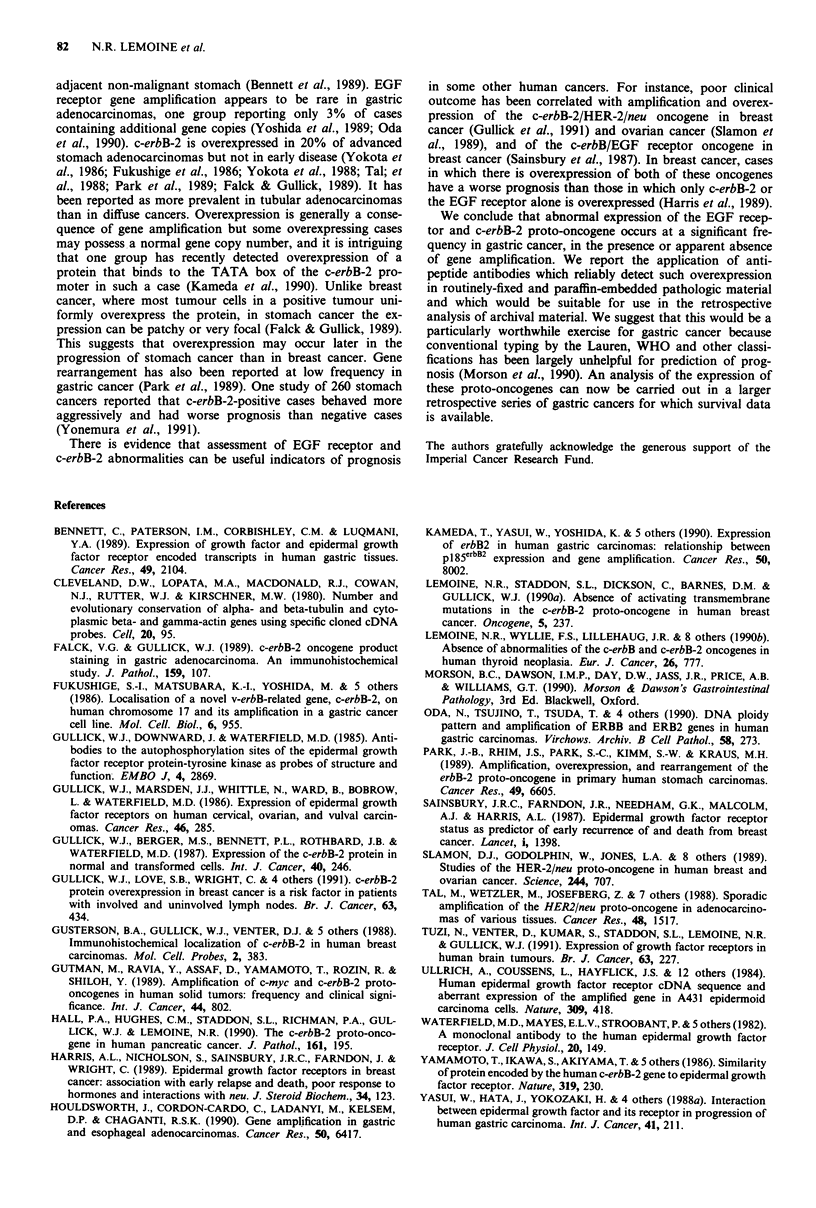

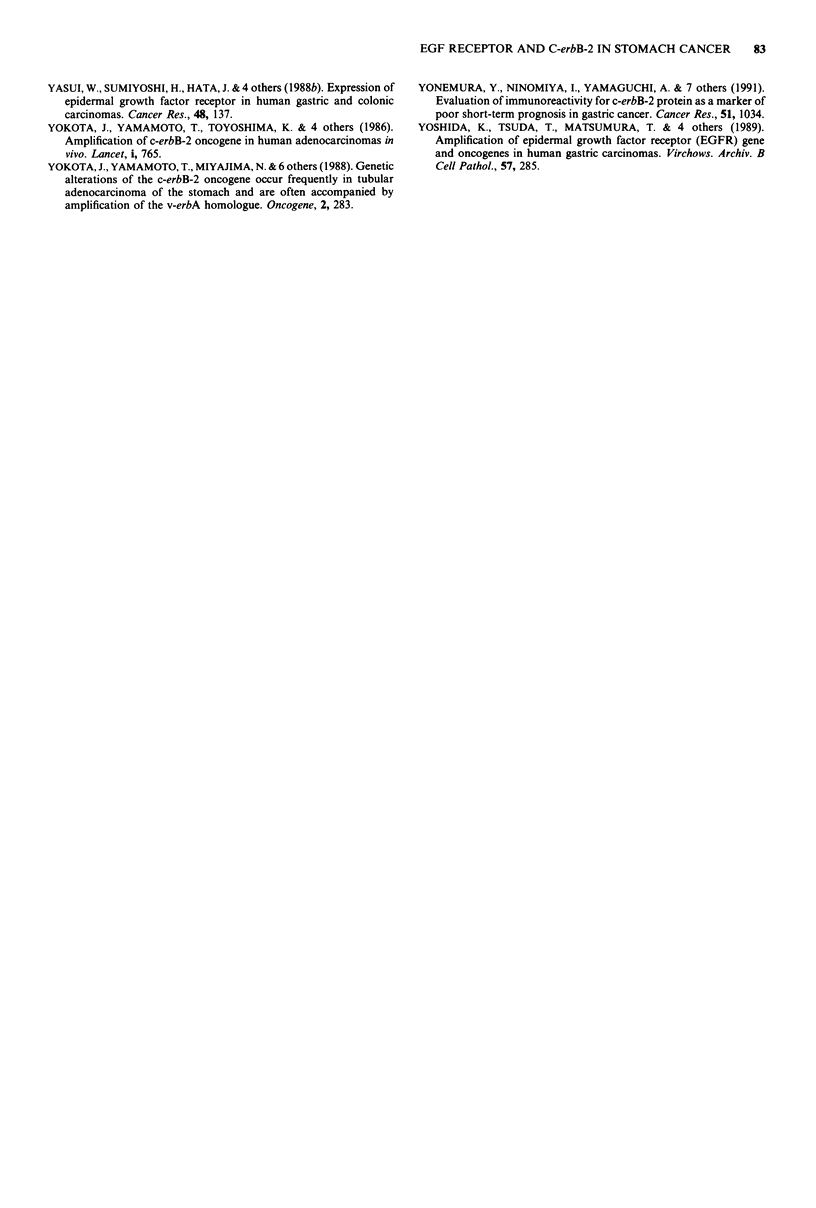


## References

[OCR_00469] Bennett C., Paterson I. M., Corbishley C. M., Luqmani Y. A. (1989). Expression of growth factor and epidermal growth factor receptor encoded transcripts in human gastric tissues.. Cancer Res.

[OCR_00475] Cleveland D. W., Lopata M. A., MacDonald R. J., Cowan N. J., Rutter W. J., Kirschner M. W. (1980). Number and evolutionary conservation of alpha- and beta-tubulin and cytoplasmic beta- and gamma-actin genes using specific cloned cDNA probes.. Cell.

[OCR_00482] Falck V. G., Gullick W. J. (1989). c-erbB-2 oncogene product staining in gastric adenocarcinoma. An immunohistochemical study.. J Pathol.

[OCR_00487] Fukushige S., Matsubara K., Yoshida M., Sasaki M., Suzuki T., Semba K., Toyoshima K., Yamamoto T. (1986). Localization of a novel v-erbB-related gene, c-erbB-2, on human chromosome 17 and its amplification in a gastric cancer cell line.. Mol Cell Biol.

[OCR_00505] Gullick W. J., Berger M. S., Bennett P. L., Rothbard J. B., Waterfield M. D. (1987). Expression of the c-erbB-2 protein in normal and transformed cells.. Int J Cancer.

[OCR_00493] Gullick W. J., Downward J., Waterfield M. D. (1985). Antibodies to the autophosphorylation sites of the epidermal growth factor receptor protein-tyrosine kinase as probes of structure and function.. EMBO J.

[OCR_00510] Gullick W. J., Love S. B., Wright C., Barnes D. M., Gusterson B., Harris A. L., Altman D. G. (1991). c-erbB-2 protein overexpression in breast cancer is a risk factor in patients with involved and uninvolved lymph nodes.. Br J Cancer.

[OCR_00499] Gullick W. J., Marsden J. J., Whittle N., Ward B., Bobrow L., Waterfield M. D. (1986). Expression of epidermal growth factor receptors on human cervical, ovarian, and vulval carcinomas.. Cancer Res.

[OCR_00521] Gutman M., Ravia Y., Assaf D., Yamamoto T., Rozin R., Shiloh Y. (1989). Amplification of c-myc and c-erbB-2 proto-oncogenes in human solid tumors: frequency and clinical significance.. Int J Cancer.

[OCR_00529] Hall P. A., Hughes C. M., Staddon S. L., Richman P. I., Gullick W. J., Lemoine N. R. (1990). The c-erb B-2 proto-oncogene in human pancreatic cancer.. J Pathol.

[OCR_00532] Harris A. L., Nicholson S., Sainsbury J. R., Farndon J., Wright C. (1989). Epidermal growth factor receptors in breast cancer: association with early relapse and death, poor response to hormones and interactions with neu.. J Steroid Biochem.

[OCR_00537] Houldsworth J., Cordon-Cardo C., Ladanyi M., Kelsen D. P., Chaganti R. S. (1990). Gene amplification in gastric and esophageal adenocarcinomas.. Cancer Res.

[OCR_00542] Kameda T., Yasui W., Yoshida K., Tsujino T., Nakayama H., Ito M., Ito H., Tahara E. (1990). Expression of ERBB2 in human gastric carcinomas: relationship between p185ERBB2 expression and the gene amplification.. Cancer Res.

[OCR_00548] Lemoine N. R., Staddon S., Dickson C., Barnes D. M., Gullick W. J. (1990). Absence of activating transmembrane mutations in the c-erbB-2 proto-oncogene in human breast cancer.. Oncogene.

[OCR_00554] Lemoine N. R., Wyllie F. S., Lillehaug J. R., Staddon S. L., Hughes C. M., Aasland R., Shaw J., Varhaug J. E., Brown C. L., Gullick W. J. (1990). Absence of abnormalities of the c-erbB-1 and c-erbB-2 proto-oncogenes in human thyroid neoplasia.. Eur J Cancer.

[OCR_00564] Oda N., Tsujino T., Tsuda T., Yoshida K., Nakayama H., Yasui W., Tahara E. (1990). DNA ploidy pattern and amplification of ERBB and ERBB2 genes in human gastric carcinomas.. Virchows Arch B Cell Pathol Incl Mol Pathol.

[OCR_00569] Park J. B., Rhim J. S., Park S. C., Kimm S. W., Kraus M. H. (1989). Amplification, overexpression, and rearrangement of the erbB-2 protooncogene in primary human stomach carcinomas.. Cancer Res.

[OCR_00575] Sainsbury J. R., Farndon J. R., Needham G. K., Malcolm A. J., Harris A. L. (1987). Epidermal-growth-factor receptor status as predictor of early recurrence of and death from breast cancer.. Lancet.

[OCR_00581] Slamon D. J., Godolphin W., Jones L. A., Holt J. A., Wong S. G., Keith D. E., Levin W. J., Stuart S. G., Udove J., Ullrich A. (1989). Studies of the HER-2/neu proto-oncogene in human breast and ovarian cancer.. Science.

[OCR_00586] Tal M., Wetzler M., Josefberg Z., Deutch A., Gutman M., Assaf D., Kris R., Shiloh Y., Givol D., Schlessinger J. (1988). Sporadic amplification of the HER2/neu protooncogene in adenocarcinomas of various tissues.. Cancer Res.

[OCR_00591] Tuzi N. L., Venter D. J., Kumar S., Staddon S. L., Lemoine N. R., Gullick W. J. (1991). Expression of growth factor receptors in human brain tumours.. Br J Cancer.

[OCR_00596] Ullrich A., Coussens L., Hayflick J. S., Dull T. J., Gray A., Tam A. W., Lee J., Yarden Y., Libermann T. A., Schlessinger J. Human epidermal growth factor receptor cDNA sequence and aberrant expression of the amplified gene in A431 epidermoid carcinoma cells.. Nature.

[OCR_00604] Waterfield M. D., Mayes E. L., Stroobant P., Bennet P. L., Young S., Goodfellow P. N., Banting G. S., Ozanne B. (1982). A monoclonal antibody to the human epidermal growth factor receptor.. J Cell Biochem.

[OCR_00609] Yamamoto T., Ikawa S., Akiyama T., Semba K., Nomura N., Miyajima N., Saito T., Toyoshima K. (1986). Similarity of protein encoded by the human c-erb-B-2 gene to epidermal growth factor receptor.. Nature.

[OCR_00614] Yasui W., Hata J., Yokozaki H., Nakatani H., Ochiai A., Ito H., Tahara E. (1988). Interaction between epidermal growth factor and its receptor in progression of human gastric carcinoma.. Int J Cancer.

[OCR_00619] Yasui W., Sumiyoshi H., Hata J., Kameda T., Ochiai A., Ito H., Tahara E. (1988). Expression of epidermal growth factor receptor in human gastric and colonic carcinomas.. Cancer Res.

[OCR_00629] Yokota J., Yamamoto T., Miyajima N., Toyoshima K., Nomura N., Sakamoto H., Yoshida T., Terada M., Sugimura T. (1988). Genetic alterations of the c-erbB-2 oncogene occur frequently in tubular adenocarcinoma of the stomach and are often accompanied by amplification of the v-erbA homologue.. Oncogene.

[OCR_00626] Yokota J., Yamamoto T., Toyoshima K., Terada M., Sugimura T., Battifora H., Cline M. J. (1986). Amplification of c-erbB-2 oncogene in human adenocarcinomas in vivo.. Lancet.

[OCR_00635] Yonemura Y., Ninomiya I., Yamaguchi A., Fushida S., Kimura H., Ohoyama S., Miyazaki I., Endou Y., Tanaka M., Sasaki T. (1991). Evaluation of immunoreactivity for erbB-2 protein as a marker of poor short term prognosis in gastric cancer.. Cancer Res.

[OCR_00639] Yoshida K., Tsuda T., Matsumura T., Tsujino T., Hattori T., Ito H., Tahara E. (1989). Amplification of epidermal growth factor receptor (EGFR) gene and oncogenes in human gastric carcinomas.. Virchows Arch B Cell Pathol Incl Mol Pathol.

